# Whole-Genome Sequences of Bacteremia Isolates of *Bordetella holmesii*

**DOI:** 10.1128/genomeA.01023-17

**Published:** 2017-09-28

**Authors:** Hervé Tettelin, Thomas A. Hooven, Xuechu Zhao, Qi Su, Lisa Sadzewicz, Luke J. Tallon, Claire M. Fraser, Adam J. Ratner

**Affiliations:** a Department of Microbiology and Immunology, Institute for Genome Sciences, University of Maryland School of Medicine, Baltimore, Maryland, USA; b Department of Pediatrics, Columbia University, New York, New York, USA; c Departments of Pediatrics and Microbiology, New York University School of Medicine, New York, New York, USA

## Abstract

*Bordetella holmesii* causes respiratory and invasive diseases in humans, but its pathogenesis remains poorly understood. We report here the genome sequences of seven bacteremia isolates of *B. holmesii*, including the type strain. Comparative analysis of these sequences may aid studies of *B. holmesii* biology and assist in the development of species-specific diagnostic strategies.

## GENOME ANNOUNCEMENT

*Bordetella holmesii* is an opportunistic pathogen that is an occasional etiology of pertussis-like respiratory illnesses and can cause invasive disease in immunocompromised individuals, particularly those with impaired splenic function (1). In the mid-1990s, the organisms previously known as the nonoxidizer-2 group were reclassified into the species *B. holmesii* (2), and the isolate from the first known invasive case, which occurred in 1983, was designated the type strain (CDC F5101, now called ATCC 51541). The majority of invasive cases have involved isolated bacteremia, but there have been sporadic reports of endocarditis/pericarditis, septic arthritis, and meningitis due to *B. holmesii* (3–11). There has been increased interest in *B. holmesii* in recent years, as improved molecular techniques have facilitated its identification (12). Several groups have reported whole-genome sequences of *B. holmesii* clinical isolates (13–15), and a detailed analysis of *Bordetella* genome evolution has been performed (16). Here, we report the genome sequences of seven additional strains (Table 1), including two complete genomes (the type strain and strain 44057, which we previously reported in draft form [13]).

**TABLE 1:** Characteristics of the sequenced *B. holmesii* strains^a^

Strain name	Genome length (bp)	No. of contigs	Accession no.	Source characteristics		
				Age (yr)	Gender (M/F)^b^	Known immunocompromising conditions
ATCC 51541^T^	3,699,674	1	CP007494	37	M	Unknown (2)
44057	3,697,138	1	CP007495	7	F	Sickle cell disease
35009	3,734,358	6	JDSK00000000	5	M	Sickle cell disease
41130	3,716,989	4	JDSC00000000	77	M	Organ transplant
70147	3,766,893	9	JDSJ00000000	73	F	Lymphoma
30539	3,762,889	9	JDFP00000000	43	F	Unknown
1058	3,727,750	4	JDTF00000000	16	F	Sickle cell disease

a All strains were isolated from blood.

b M, male; F, female.

The whole-genome sequences were determined using a hybrid approach that combined HiSeq 2000 and Pacific Biosciences RS technologies. Outputs and metrics for individual sequencing runs are available in the Sequence Read Archive. Assembly was performed using Hierarchical Genome Assembly in SMRT (HGap version 1.4). The resulting assemblies ranged from 1 to 9 contigs, with genome sizes of approximately 3.7 Mbp per genome. The GC contents were 62.6 to 62.8%, consistent with those of previously reported *B. holmesii* genomes. Annotation was performed using the IGS Prokaryotic Annotation Engine, as previously described (17).

These sequences will be useful for comparative analyses with other *B. holmesii* strains and other bordetellae. Specific features, including predicted siderophore biosynthesis genes, virulence regulatory systems, and the previously noted toluene-4-monoxygenase system (13), are present and may be useful for studies of pathogenesis and for the design of *B. holmesii*-specific detection strategies.

### Accession number(s).

These whole-genome sequences have been deposited in GenBank under the accession numbers that appear in Table 1.
